# Ferroptosis: A Novel Therapeutic Target for Ischemia-Reperfusion Injury

**DOI:** 10.3389/fcell.2021.688605

**Published:** 2021-08-10

**Authors:** Yunqing Chen, Hongyan Fan, Shijun Wang, Guanmin Tang, Changlin Zhai, Liang Shen

**Affiliations:** ^1^Department of Infectious Disease, The Affiliated Hospital of Jiaxing University, Jiaxing, China; ^2^Department of Cardiology, The Affiliated Hospital of Jiaxing University, Jiaxing, China

**Keywords:** ferroptosis, Ischemia/reperfusion injury, iron, lipid peroxidation, therapeutic target

## Abstract

Ischemia-reperfusion (I/R) injury is a major cause of cell death and organ damage in numerous pathologies, including myocardial infarction, stroke, and acute kidney injury. Current treatment methods for I/R injury are limited. Ferroptosis, which is a newly uncovered type of regulated cell death characterized by iron overload and lipid peroxidation accumulation, has been investigated in various diseases. There is increasing evidence of a close association between ferroptosis and I/R injury, with ferroptosis frequently identified as a new therapeutic target for the management of I/R injury. This review summarizes the current status of ferroptosis and discusses its relationship with I/R injury, as well as potential treatment strategies targeting it.

## Introduction

Ischemia-reperfusion (I/R) injury results from an initial restriction of blood supply to an organ or tissue followed by the restoration of perfusion, which leads to morbidity and mortality in a wide range of pathologies. I/R injury occurs in many organs, including the heart, brain, kidney, liver, and lung, and its mechanisms include oxidative stress, inflammation, mitochondrial dysfunction, calcium overload, microvascular dysfunction, and the activation of cell death pathways (Eltzschig and Eckle, 2011). While significant advances have been made in the treatment of I/R injuries, therapy for this condition remains to be a significant challenge (Pantazi et al., 2016); therefore, it is important to explore new therapeutic targets to manage I/R injury.

Ferroptosis, an iron-dependent regulated cell death (Dixon et al., 2012), has recently been discovered in a variety of pathologies and proposed as a novel therapeutic strategy for various diseases, including I/R injury (Friedmann Angeli et al., 2014; Fang et al., 2019). In this review, we summarize the mechanisms of ferroptosis and discuss its process as a potential therapeutic target for I/R injury.

## Overview of Ferroptosis

Dolma et al. (2003) discovered erastin, a new compound that exerted a selectively lethal effect on BJeLR cells expressing the engineered mutant RAS oncogene, but this mode of cell death differed from any other modes encountered before. Subsequent studies further demonstrated that iron chelators suppressed erastin-induced cell death while Ras-selective lethal small molecule (RSL) initiated it (Yang and Stockwell, 2008). Dixon et al. (2012) termed this unique cell death type ferroptosis. Ferroptosis differs from other types of regulated cell deaths (RCD), including apoptosis, autophagy, and necroptosis in morphology, biochemistry, and genetics (Table 1). It is mainly characterized by the shrinking of the mitochondria with an increased mitochondrial membrane density and the breakdown of mitochondrial crista, all while the morphology of the nucleus remains unchanged.

**TABLE 1:** Characteristics of the different types of cell death.

Type of cell death	Morphological features	Biochemical characteristics	Regulation
Ferroptosis	Small mitochondria, increased mitochondrial membrane density, breakdown of cristae	Iron accumulation and lipid peroxidation	Positive regulator: ACSL4, Hmox1, NCOA4 Negative regulator: GPX4, Nrf2, HSPB1, SLC7A11, FSP1
Apoptosis	Cell contraction, membrane blebbing, chromatin condensation, and apoptotic bodies formation	DNA fragmentation	Positive regulator: Bax family, p53 Negative regulator: Bcl-2 family
Autophagy	Accumulation of double-membrane vesicles	Lysosomal activation	Positive regulator: ATG5, ATG7, Beclin 1 Negative regulator: mTOR
Necroptosis	Cytoplasm and organelles swelling, plasma membrane breakup	ROS production, damage-associated molecular patterns (DAMPs) liberation	Positive regulator: RIPK1, RIPK3, MLKL Negative regulator: Flotillin, syntenin-1

Ferroptosis can be induced by several chemical compounds and drugs, including erastin, RSL 3, sulfasalazine, and sorafenib; suppressed by peroxidation inhibitors, iron chelators, and antioxidants (Stockwell et al., 2017); regulated by numerous genes, such as nuclear receptor coactivator 4 (NCOA4), heme oxygenase 1 (Hmox1), acyl-CoA synthetase long-chain family member 4 (ACSL4), glutathione peroxidase 4 (GPX4), cystine/glutamate antiporter solute carrier family 7 member 11 (SLC7A11), nuclear factor erythroid 2-related factor 2 (Nrf2), and ferroptosis suppressor protein (FSP1); and modulated by multiple cellular metabolic pathways, like iron metabolism, amino acid metabolism, and lipid metabolism (Li J. et al., 2020; Ju et al., 2021; Yan et al., 2021).

## Mechanisms of Ferroptosis

**FIGURE 1:**
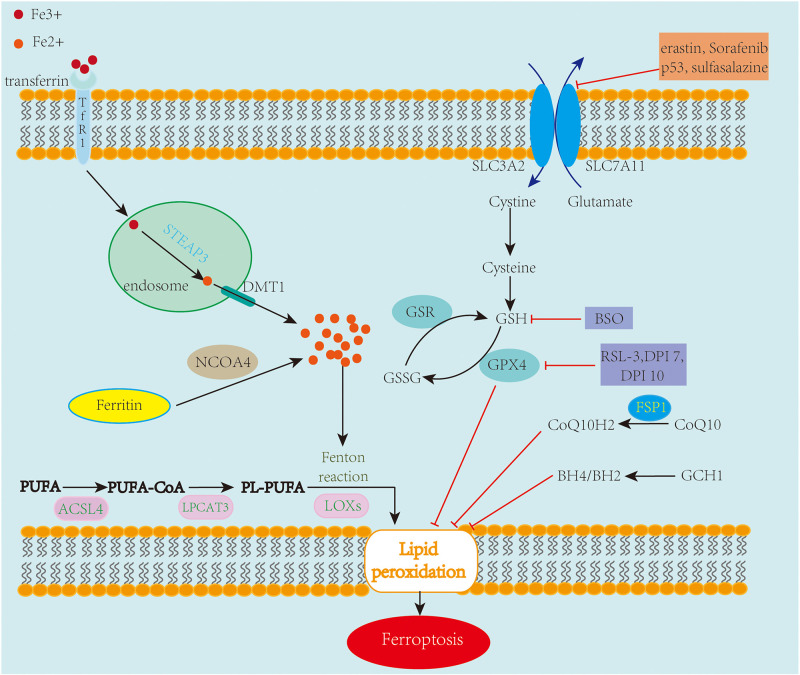
An overview of the mechanisms of ferroptosis. Ferroptosis is an iron-dependent cell death characterized by the accumulation of lipid peroxidation. Free iron ions accumulate and catalyze the Fenton reaction, leading to the formation of lipid peroxides and eventually ferroptosis. The System xc^–^/GSH/GPX4 axis is regarded as the primary pathway involved in ferroptosis. Inhibiting SLC7A11 and GPX4 leads to the accumulation of lipid peroxidation, causing ferroptotic cell death. Also, FSP1-CoQ10 and GCH1-BH4/BH2 are two GPX4-independent pathways in the inhibition of ferroptosis.

Ferroptosis mechanisms are complex, involving a range of signaling molecules and metabolic pathways (Figure 1). The main metabolic pathways in the pathogenesis of ferroptosis are discussed below.

### Amino Acid Metabolism and Ferroptosis

System xc^–^ is a cystine/glutamate antiporter containing two subunits, SLC7A11 and SLC3A2. Its main function is interchanging cystine and glutamate across the cell membrane (Sato et al., 1999). Once taken up by system xc^–^ and transported into the cell, cystine is reduced to cysteine, which is an essential amino acid for the biosynthesis of glutathione (GSH). GSH, a potent antioxidant that enhances the anti-lipid peroxidation activity of GPX4, which is a member of glutathione peroxidases (GPxs), is regarded as the key regulator of ferroptosis (Yang et al., 2014). The System xc^–^/GSH/GPX4 axis is considered the primary pathway involved in ferroptosis. Numerous investigations have been conducted on chemical compounds and genes targeting this amino acid metabolism pathway.

Erastin, a RSL compound, inhibits system xc^–^, causing a depletion in GSH, which subsequently suppresses the activity of GPX4 and promotes the formation of lipid ROS and ferroptosis (Dixon et al., 2012). BRCA1-associated protein 1 (BAP1) and OTU deubiquitinase, ubiquitin aldehyde-binding 1 (OTUB1) are also involved in ferroptosis via the regulation of the expression of system xc− (Zhang et al., 2018; Liu et al., 2019). RSL3, another RSL compound, inhibits GPX4 directly without depleting GSH, and DPI7 and DPI10 likewise act directly on GPX4 to induce ferroptosis (Yang et al., 2014). Like erastin, Sorafenib, a multikinase inhibitor approved for hepatic cancer treatment (Zhang et al., 2013), causes ferroptosis by blocking GSH synthesis (Lachaier et al., 2014), and Buthionine sulfoximine (BSO), which has been studied as an adjunct treatment for cancer, stimulates ferroptosis by reducing GSH levels. Tumor suppressor gene, p53, also plays an important role during ferroptosis, inhibiting system xc^–^ by downregulating the expression of SLC7A11, thus impacting the activity of GPX4, which causes an increase in lipid peroxidation and ferroptotic cell death (Jiang et al., 2015). FIN56 depletes CoQ10 via the squalene synthase activity (SQS)-mevalonate pathway and promotes GPX4 degradation, resulting in ferroptosis too (Shimada et al., 2016).

### Iron Metabolism and Ferroptosis

Iron homeostasis is essential for many biological processes and cell viability. However, both iron overload and deficiency can lead to diverse diseases. Ferroptosis is triggered by excessive iron, and an excess amount of iron causes lipid peroxidation and cell death. Although the detailed mechanisms between iron metabolism and ferroptosis remain to be substantially elucidated, there is no doubt that iron metabolism plays a crucial role in ferroptosis.

Fe^3+^ is transported into cells by transferrin receptor 1 (TfR1), where it is converted by the six-transmembrane epithelial antigen of the prostate 3 (STEAP3) to Fe^2+^, which is then released from endosomes through the divalent metal transporter 1 (DMT1). The released Fe^2+^ is stored in an unstable iron pool and ferritin and exported by ferroportin-1 (FPN1) (Chen et al., 2020). If the balance between iron absorption, utilization, and recycling is interrupted, free iron ions may accumulate and catalyze the Fenton reaction, resulting in the formation of lipid ROS and ferroptosis. Silencing the gene encoding TfR1 inhibits erastin-induced ferroptosis (Gao et al., 2015), whereas depleting FPN1 increases cell sensitivity to ferroptosis (Geng et al., 2018).

NCOA4 is a selective cargo receptor for the degradation of ferritin. It maintains iron homeostasis by modulating ferritinophagy (Dowdle et al., 2014; Mancias et al., 2014). Suppressing NCOA4 abolishes the accumulation of reactive iron and ROS and eventually ferroptotic cell death; on the other hand, overexpressed NCOA4 promotes ferroptosis and augments ferritin degradation (Gao et al., 2016; Hou et al., 2016). Knocking down Poly-(rC)-binding protein 1 (PCBP1), a cytosolic iron chaperone that carries iron to ferritin, increases the amount of labile iron, promoting ferroptosis in the liver (Protchenko et al., 2021). HSPB1, a member of small heat shock proteins (HSPs), a class of functionally related stress proteins, can inhibit iron uptake (Chen et al., 2006), and its overexpression suppresses erastin-induced ferroptosis (Sun et al., 2015). Hmox1, which is a critical enzyme in heme catabolism that breaks downs heme into iron, biliverdin, and carbon monoxide, could also be crucial in ferroptosis. Research has revealed that Hmox1 knockout mice have an iron overload in the liver and kidney, and inhibiting Hmox1 with ZnPP reverses doxorubicin-induced ferroptosis in the myocardium (Fang et al., 2019). However, in renal proximal tubule cells, silencing Hmox1 enhances erastin- or RSL3-induced ferroptosis (Adedoyin et al., 2018), suggesting that the role of Hmox1 in ferroptosis might be context-dependent.

### Lipid Metabolism and Ferroptosis

Lipid peroxidation is a key feature of ferroptosis. Polyunsaturated fatty acids (PUFAs), which are components of the cell membrane, are the most susceptible lipids to peroxidation during ferroptosis. PUFAs are converted to PUFA-CoAs by ACSL4, an enzyme that participates in phospholipid metabolism. The obtained PUFA-CoAs are esterified by lysophosphatidylcholine acyltransferase 3 (LPCAT3) to form PL-PUFAs, which are then oxidized by lipoxygenases (LOXs) into lipid hydroperoxides, which serve as ferroptotic signals (Li and Li, 2020). Downregulating the expression of ACSL4 and LPCTA3 contributes to the inhibition of ferroptosis, with ACSL4 thought to be more efficient than LPCTA3 during this process (Dixon et al., 2015; Doll et al., 2017). LOXs represent a family of iron-containing enzymes that catalyze the dioxygenation of PUFAs. LOXs, particularly 12/15-LOX, play a central role in lipid peroxidation and ferroptosis (Yang et al., 2016; Kagan et al., 2017). Phosphatidylethanolamine-binding protein 1 (PEBP1), a small scaffold protein inhibitor of protein kinase cascades, forms complexes with 15-LOX to promote ferroptosis (Wenzel et al., 2017). In contrast to PUFAs, monounsaturated fatty acids (MUFAs) exert anti-ferroptotic effects by inhibiting lipid peroxidation. One investigation showed that the overexpression of stearoyl-CoA desaturase1 (SCD1), an essential enzyme in the biosynthesis of MUFAs, in ovarian cancer cells suppresses ferroptosis (Tesfay et al., 2019).

### Other Pathways Involved in Ferroptosis

With research on ferroptosis expanding rapidly, new regulatory pathways independent of GPX4 have been uncovered recently. FSP1 was first described as a p53-responsive gene; however, two studies most recently revealed simultaneously that FSP1 is an effective ferroptosis suppressor, protecting cells from ferroptosis by modulating CoQ10 (Bersuker et al., 2019; Doll et al., 2019). Using a CRISPR-mediated whole-genome activation screen technique, guanosine triphosphate cyclohydrolase 1 (GCH1) was found to participate in the regulation of ferroptosis. Upregulating or silencing GCH1 renders cancer cells resistant or sensitive, respectively, to ferroptosis by controlling antioxidant BH4 (Kraft et al., 2020). Hence, targeting the GCH1/BH4 axis and FSP1/CoQ10 axis could provide novel ideas for drug discovery.

## Ferroptosis and I/R Injury

**FIGURE 2:**
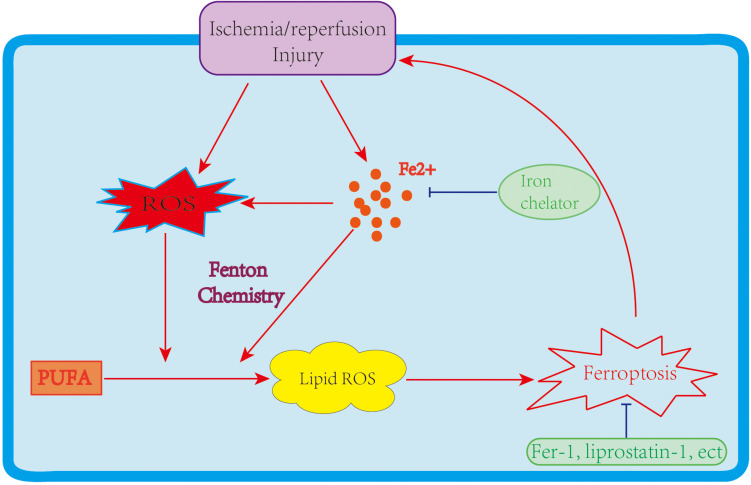
The link between ferroptosis and I/R injury. An I/R injury causes iron accumulation and ROS generation, leading to lipid peroxidation and ferroptosis. Ferroptosis further enlarges I/R injuries; however, iron chelators and ferroptosis inhibitors improve the damage caused by I/R.

Two major mechanisms of I/R injury are oxidative stress and cell death. During oxidative stress, a “burst” of ROS is produced. ROS generation depends primarily on the mitochondrial respiratory chain and NADPH oxidase (NOX) family. As noted above, PUFAs are susceptible to ROS, leading to lipid peroxidation and ferroptosis. Cell death could be a key contributor to I/R injury. In the past few decades, various types of cell death, including apoptosis, necrosis, and autophagy-associated cell deaths, have been identified during I/R injury (Logue et al., 2005; Gottlieb, 2011), with therapeutic methods relating to these cell death types also investigated. Still, the treatment of I/R injury remains significantly challenging. Recently, ferroptosis has raised fresh hope and impetus in the fight to treat I/R injury. Mounting data indicate that ferroptosis participates in I/R, which makes targeting ferroptosis a potentially feasible and effective approach to reducing I/R injury (Figure 2). This section discusses the role of ferroptosis in I/R injury in different organs and the likely therapeutic strategy to slow down the process of I/R injury (Table 2).

**TABLE 2:** Potential therapeutic strategy for I/R injury.

Reagents	Mechanism	Diseases	References
Ferrostatin-1	Inhibit lipid peroxidation	myocardial, cerebral, and liver I/R injury	Tuo et al., 2017; Fang et al., 2019; Yamada et al., 2020
Dexrazoxane	Chelation of iron	myocardial I/R injury	Fang et al., 2019
Deferoxamine	Reduce intracellular iron	myocardial, cerebral, liver, and testicular I/R injury	Hanson et al., 2009; Li et al., 2018; Yamada et al., 2020; Tu et al., 2021
Liproxstatin-1	Inhibit lipid peroxidation	myocardial, cerebral, liver, intestinal, pulmonary, and testicular I/R injury	Friedmann Angeli et al., 2014; Tuo et al., 2017; Li et al., 2018; Feng et al., 2019; Li Y. et al., 2019; Xu et al., 2020
Galangin	Increase expression of GPX4	cerebral I/R injury	Guan et al., 2021
Carvacrol	Increase expression of GPX4	cerebral I/R injury	Guan et al., 2019
16–86	Inhibit lipid peroxidation	renal I/R injury	Linkermann et al., 2014
Quercetin	Inhibit lipid peroxidation	renal I/R injury	Wang et al., 2021
α-tocopherol	Inhibit lipid peroxidation	liver I/R injury	Yamada et al., 2020
Rosiglitazone	Inhibit ACSL4	intestinal and pulmonary I/R injury	Li Y. et al., 2019; Xu et al., 2020

*I/R, ischemia/reperfusion; GPX4, glutathione peroxidase 4; ACSL4, acyl-CoA synthetase long-chain family member 4.*

### Ferroptosis and Myocardial I/R Injury

Compared to sham-operated mice, mice subjected to myocardial I/R have been shown to have significantly higher levels of cardiac Ptgs2 expression, with pretreatment with ferrostatin-1 (Fer-1, a ferroptosis inhibitor) or dexrazoxane (DXZ, an iron chelator) preventing I/R-induced elevations in myocardial enzymes and reducing myocardial infarct size (Fang et al., 2019). Similar to Fer-1 and DXZ, liproxstatin-1 treatment shrinks myocardial infarct size, and, additionally, it maintains mitochondrial structure and function after myocardial I/R by decreasing VDAC1 levels and restoring GPX4 levels (Feng et al., 2019). Inhibiting glutaminolysis, an essential component of ferroptosis, also attenuates I/R-triggered heart injury (Gao et al., 2015).

A recent investigation showed that ponatinib or deferoxamine suppresses necroptosis or ferroptosis in myocardial I/R, and combination therapy further decreased the myocardial infarct size (Tu et al., 2021). However, deferoxamine administered to patients with ST-elevated myocardial infarction via primary percutaneous coronary intervention (PPCI) before reperfusion does not reduce the infarct size (Chan et al., 2012). These results indicate that co-treatment targeting the different types of cell death might be an effective therapeutic strategy for I/R injury.

Ferroptosis is involved in diabetes-related myocardial I/R injury as well. Fer-1 reduces myocardial injury in a rat I/R model and cell injury in H9c2 cells during diabetes-related myocardial I/R, providing a therapeutic reagent for myocardial ischemia in diabetic patients (Li W. et al., 2020).

Heart transplantation is another procedure blighted by myocardial I/R injury, with Fer-1 lessening cardiomyocyte death and blocking neutrophil recruitment following heart transplantation; therefore, targeting ferroptosis could provide therapeutic strategies for heart transplant recipients (Li W. et al., 2019).

### Ferroptosis and Cerebral I/R Injury

Iron overload has been proposed as the key mediator of neuronal damage and I/R-related death. Deferoxamine treatment immediately after brain reperfusion decreases infarct volume (Hanson et al., 2009), and suppressing tau, a protein of Alzheimer’s disease, protects against cerebral ischemia-reperfusion injury in young mice via ferroptosis inhibition, with ferroptosis inhibitors, such as liproxstatin-1 and Fer-1, reviving the protection provided by tau knockout in older mice (Tuo et al., 2017). Galangin cloud improves the learning ability and memory in gerbils after I/R injury by inhibiting ferroptosis through increased SLC7A11 and GPX4 expressions (Guan et al., 2021), suggesting that the protective effect of galangin on cerebral I/R injury is affected through the inhibition of ferroptosis. Carvacrol, a monoterpenoid phenol, also protects hippocampal neurons against I/R injury by increasing the expression of GPX4 (Guan et al., 2019). All these results support the theory that ferroptosis actively participates in cerebral I/R injury, pointing to ferroptosis as a potential target for cerebral ischemia-reperfusion injury treatment.

### Ferroptosis and Renal I/R Injury

Renal tubular cell death is a crucial factor in the pathogenesis of kidney I/R injury. A novel third-generation ferrostatin, 16–86, protects mice from functional acute renal failure and structural organ damage after renal I/R injury. Additionally, combining this substance with necrostatins and compounds that inhibit mitochondrial permeability transition further potentiates the protective effect of 16–86 on I/R injury (Linkermann et al., 2014). Necroptosis might not be the predominant pathway through which necrosis in tubular cells is regulated, with ferroptosis-mediated necrosis identified as the main pathophysiology in renal I/R injury (Linkermann et al., 2014).

MicroRNAs (miRNAs) are a class of small non-coding RNAs that function in various biological processes involving cell death. In I/R-induced renal injury, miR-182-5p and miR-378-3p are upregulated, which promotes the activation of ferroptosis through the downregulation of GPX4 and SLC7A11 (Ding et al., 2020), indicating that ferroptosis may be regulated by multiple I/R-related miRNAs and targeting these miRNAs could provide novel therapeutic methods to suppress ferroptosis and I/R injury.

Quercetin, a natural flavonoid, has recently been identified as an inhibitor of ferroptosis because it decreases the levels of malondialdehyde and lipid peroxidation and increases GSH levels; therefore, quercetin could be a novel treatment for renal I/R injury (Wang et al., 2021).

### Ferroptosis and Liver I/R Injury

In mice with hepatic I/R injury, liproxstatin-1 treatment decreases the ratio of ALT/AST and percentage of necrotic areas, indicating that ferroptosis inhibitors mitigate I/R-caused liver injuries (Friedmann Angeli et al., 2014).

Hepatic I/R injuries are a major problem in liver transplantation. High ferritin levels in donors are an important predictor of liver damage after liver transplantation (Yamada et al., 2020). Fer-1 or α-tocopherol prevents liver damage, lipid peroxidation, and Ptgs2 upregulation in a murine model of hepatic I/R injury, and deferoxamine attenuates hepatic I/R injuries, pointing to ferroptosis as a potential treatment target for liver I/R injury (Yamada et al., 2020).

### Ferroptosis and Other I/R Injuries

In intestinal I/R, ferroptosis occurs in the early stage of reperfusion. Treatment with liproxstatin-1 alleviates intestinal I/R injury by increasing GPX4 expression and reducing Ptgs2 expression and lipid peroxidation. Pretreatment with rosiglitazone, an inhibitor of ACSL4, also improves intestinal I/R injury through regulating GPX4 and Ptgs2 expressions, implying that targeting ferroptosis could be an effective therapeutic approach for intestinal I/R injury (Li Y. et al., 2019).

Lung I/R increases iron concentration, lipid peroxidation accumulation, and the expression of GPX4 and ACSL4. Pretreatment with liproxstatin-1 or rosiglitazone inhibits ferroptosis and improves pulmonary I/R injury (Xu et al., 2020).

In oxygen-glucose deprivation and reoxygenation (OGD/R)-induced I/R injury in TM4 cells, iron and lipid peroxidation levels are elevated, mitochondrial size decreases, and membrane density increases; however, OGD/R-caused cell death is obstructed by lipid peroxidation inhibitors, liproxstatin-1, and the iron chelator, deferoxamine. These results provide a novel insight into the inhibition of ferroptosis in the treatment of testicular I/R injury (Li et al., 2018).

## Conclusion and Perspective

I/R-instigated cell death results in drastic impairments to organ functions. Preventing and reducing cell death is crucial to the improvement and preservation of organ functions during I/R injury. Ferroptosis, a newly uncovered type of cell death dependent on iron accumulation, is increasingly garnering attention and is emerging as a potential therapeutic target for I/R injury.

However, the use of iron chelators to treat I/R injuries in the past few decades has produced unsatisfactory results (Lesnefsky et al., 1990; Nakamura et al., 1990; Judy et al., 1994; Chan et al., 2012), a disappointment likely due to several factors, including the dosage, cytotoxicity, and organ specificity of iron chelators. Besides, the cell death pathways responsible for the pathogenesis of I/R injury are complex: multiple cell death types, including apoptosis, autophagy, and necroptosis, are involved in the process of I/R injury (Hamacher-Brady et al., 2006; Jun et al., 2020). Although comprehensively linking ferroptosis to other RCDs remains a tall order, increasing evidence shows a crosstalk between ferroptosis and other cell death types. In mouse embryonic fibroblasts, autophagy stimulates ferroptosis by degrading ferritin (Hou et al., 2016), affirming ferroptosis as an autophagic cell death process (Gao et al., 2016). Ferroptosis and necroptosis are also regarded as alternatives and are intertwined in murine renal I/R injury (Muller et al., 2017). Moreover, oxytosis, a form of non-apoptotic RCD, shows the same characteristics as ferroptosis and can be suppressed by iron chelators (Tan et al., 2001). Furthermore, ferroptosis and apoptosis can be regulated by the same modulators, such as p53, and ferroptotic agents induce endoplasmic reticulum stress and promote apoptosis through the CHOP/PUMA pathway (Hong et al., 2017). All this evidence suggests that there is a close link between ferroptosis and other cell death patterns. Yet, the cell death type that dominates I/R-induced injury has still to be determined. Therefore, further studies must be conducted to specifically identify the contributions of different cell death pathways to the development of I/R injury. Since many cell death types contribute to the progression of I/R injury, combination treatments targeting different cell death pathways are potentially the most effective strategies to limiting the severity of I/R injury.

In reality, ferroptosis participating in the pathophysiology of I/R injury is not surprising, given that I/R injuries generate a “burst” of ROS, which is one of the main causes of ferroptosis. However, it is unclear yet whether ferroptosis acts as a response to I/R injuries or is the stimulus that directly enlarges I/R injuries. Further studies must be performed to explore the exact relationship between ferroptosis and I/R injury. Nevertheless, accumulating evidence indicates that targeting ferroptosis could suppress I/R injury. Most recently, several flavonoids were discovered as protectors of I/R injury via inhibiting ferroptosis. For example, baicalin was found to prevent myocardial I/R injury through suppressing ACSL4-mediated ferroptosis (Fan et al., 2021). Cyanidin-3-glucoside, another flavonol, was demonstrated to attenuated myocardial I/R injury through ferroptosis (Shan et al., 2021). Besides, kaempferol was also found to protect against oxygen-glucose deprivation/reperfusion-induced neuronal injury by inhibiting ferroptosis (Yuan et al., 2021). As mentioned above, mitochondrial dysfunction is one of the major mechanisms of injuries caused by I/R, mitochondrial ROS suppressor or scavenger, such as MitoTEMPO or XJB-5-131, might reverse the function of the mitochondria and inhibit ferroptosis, ultimately providing therapeutic strategies for I/R injury (Ni et al., 2016; Zhao et al., 2020). Additionally, as a form of RCD, ferroptosis triggers neutrophil recruitment through the release of damage-associated molecular patterns (DAMPs), and then stimulates innate immune receptors such as TLRs, eventually leading to the initiation of inflammation (Li W. et al., 2019), another main mechanism of I/R injury. Thus, ferroptosis inhibitors might simultaneously ameliorate inflammation, which needs to be further investigated.

Currently, the protective function of ferroptosis suppressors is limited to animal studies; how these results translate into clinical situations must yet be determined. Without a doubt, other factors and pathways regulating ferroptosis will be discovered, given how rapidly the scope of research on this recently unearthed cell death type is expanding. Targeting these genes and pathways could provide novel treatment methods for I/R injury, which, incidentally, also requires further in-depth investigations.

In conclusion, although ferroptosis is still in the early period of what should become a riveting research route, it appears to play a vital role in the pathogenesis of various I/R injuries. Like the many evaluations that are still to be performed, its mechanism must be explored further. Still, indications are that suppressing ferroptosis is potentially an effective therapeutic strategy for I/R injury.

## Author Contributions

LS: conceptualization. YC: writing. HF: software. SW: supervision. GT: funding acquisition. CZ: editing. All authors have read and approved the content of the manuscript.

## Conflict of Interest

The authors declare that the research was conducted in the absence of any commercial or financial relationships that could be construed as a potential conflict of interest.

## Publisher’s Note

All claims expressed in this article are solely those of the authors and do not necessarily represent those of their affiliated organizations, or those of the publisher, the editors and the reviewers. Any product that may be evaluated in this article, or claim that may be made by its manufacturer, is not guaranteed or endorsed by the publisher.
